# Characterization of the TCRβ repertoire of peripheral MR1-restricted MAIT cells in psoriasis vulgaris patients

**DOI:** 10.1038/s41598-023-48321-z

**Published:** 2023-11-28

**Authors:** Maja Jirouš Drulak, Zvonimir Grgić, Vera Plužarić, Marija Šola, Teuta Opačak-Bernardi, Barbara Viljetić, Kristina Glavaš, Maja Tolušić-Levak, Vlatka Periša, Martina Mihalj, Mario Štefanić, Stana Tokić

**Affiliations:** 1 Department of Medical Chemistry, Biochemistry and Clinical Chemistry, Faculty of Medicine, Josip Juraj Strossmayer University of Osijek, Osijek, Croatia; 2 Department of Laboratory Medicine and Pharmacy, Faculty of Medicine, Josip Juraj Strossmayer University of Osijek, Osijek, Croatia; 3 Department of Dermatology and Venerology, University Hospital Osijek, Osijek, Croatia; 4 Department of Transfusion Medicine, University Hospital Osijek, Osijek, Croatia; 5 Department of Histology and Embryology, Faculty of Medicine, Josip Juraj Strossmayer University of Osijek, Osijek, Croatia; 6 Department of Internal Medicine and History of Medicine, Faculty of Medicine, Josip Juraj Strossmayer University of Osijek, Osijek, Croatia; 7 Department of Hematology, Clinic of Internal Medicine, University Hospital Osijek, Osijek, Croatia; 8 Department of Physiology and Immunology, Faculty of Medicine, Josip Juraj Strossmayer University of Osijek, Osijek, Croatia; 9 Department of Nuclear Medicine and Oncology, Faculty of Medicine, Josip Juraj Strossmayer University of Osijek, Osijek, Croatia

**Keywords:** Immunology, Molecular biology, Diseases

## Abstract

Psoriasis vulgaris (PV) is an inflammatory skin disease largely driven by aberrant αβT cells. Mucosal-associated invariant T (MAIT) cells, which constitute the largest circulating innate-like αβT cell community in human adults, are characterized by a semi-invariant TCRVα7.2 receptor and MR1-restricted affinity toward microbial metabolites. Limited MAIT TCRα diversity is complemented by a more variable TCRβ repertoire, but its footprint in the MAIT repertoire of PV patients has never been tested. Here, we used bulk TCRSeq, MiXCR, VDJTools, and Immunarch pipelines to decipher and compare TCRβ clonotypes from flow-sorted, peripheral TCRVα7.2^+^MR1-5-OP-RU-tet^+^MAIT cells from 10 PV patients and 10 healthy, matched controls. The resulting TCRβ collections were highly private and individually unique, with small public clonotype content and high CDR3β amino acid length variability in both groups. The age-related increase in the ‘hyperexpanded’ clonotype compartment was observed in PV, but not in healthy MAIT repertoires. The TCRβ repertoires of PV patients were also marked by skewed TRBV/TRBJ pairing, and the emergence of PV-specific, public CDR3β peptide sequences closely matching the published CDR3β record from psoriatic skin. Overall, our study provides preliminary insight into the peripheral MAIT TCRβ repertoire in psoriasis and warrants further evaluation of its diagnostic and clinical significance.

## Introduction

Psoriasis vulgaris (PV) is a chronic, inflammatory skin disease mediated by the aberrantly activated immune cells. The peripheral T cell compartment constitutes a significant segment of the inflammatory infiltrate that drives the characteristic epithelial and vascular skin remodeling, largely through the release of IL-17, INF-γ and TNF-α cytokines^1–3^. Together with conventional members of the αβ T cell receptor (TCRαβ) expressing family, affected skin harbours their innate-like T cell counterparts^4^, including the innate-like T cell fraction of mucosal-associated invariant T (MAIT) cells. Human MAIT cells make up 1-10% of all blood T cells and are the largest source of the IL-17 cytokine within the circulating CD8^+^ T lymphocyte community^5^. Their murine peers are recruited to damaged skin and sustain wound healing^6,7^, but the extent to which circulating MAIT cells are associated with human skin lesions is largely unknown.

Canonical human MAIT lymphocytes respond to a variety of commensal and pathogenic microbes, in an IL-1-, IL-18-, and antigen-dependent manner, recognizing riboflavin-derived metabolites in a complex with the MHC Class I-like molecule MR1^8–11^. In doing so, MAIT cells express the semi-invariant TCRVα7.2 receptor with a conserved TCRα chain encoded by the V gene segment TRAV1-2, commonly juxtaposed to the J gene variant TRAJ33, TRAJ20, or TRAJ12^8,12,13^. The composition of the TCRβ chain provides greater variability within the MAIT TCR repertoire, although the TRBV6 and TRBV20 building segments are most often used^8,10,14^.

In contrast to the seemingly limited MAIT TCR repertoire diversity, novel evidence supports significant MAIT cell heterogeneity^15–17^, including clonal and transcriptional variability across tissues and individuals^10,18,19^. Older age, activation modalities and past antigen exposure can also shape MAIT TCR diversity^20^. Variations in classical MAIT TCRαβ rearrangements are, moreover, found among rare MR1-restricted MAIT cells with TRAV1-2-negative TCRs^21,22^, which exhibit diverse TRAV and TRBV usage and bind selectively to both classic ribityl-based (5-OP-RU), and non-classic, folate-derived^15,23^ or self-reactive MR1-ligands^24^. Furthermore, MAIT lymphocytes recognize diverse microbial^25–31^ pathogens (reviewed in Ref.^32^) and demonstrate the ability to adapt their immune response by expanding antigen-specific TCRβ clones^30^. In line, versatile MAIT TCRβ chain usage was shown to facilitate differential antigen recognition^15,33^, and aids MAIT cells to fine-tune their immune responses against various ligands^34,35^, supporting a link between the MAIT TCRβ architecture and their roles in infection^36^ and autoimmunity^37^. Hence, defining MAIT TCRβ motifs may prove useful to infer TCR variants of relevance to the pathogenesis or diagnosis of immune-mediated diseases such as psoriasis.

Previous studies addressing TCRβ repertoire diversity in the context of psoriasis have been conducted in whole skin biopsy samples^38–40^, total peripheral mononuclear cells^41^ and sorted cutaneous CD4^+^ T cell effector and regulatory T cells^42^, CD8^+^^42,43^ or CD45^+^^44^ T lymphocytes, providing little insight into the TCRβ clonotype diversity of MR1-restricted MAIT cells in PV. The MAIT TCR repertoire in healthy donors was most recently examined in single-cell TCRSeq settings, providing evidence for highly private, individually unique MAIT CDR3β sequences in the periphery^18^, while clonally expanded MAIT repertoire was primarily demonstrated in bone marrow, spleen and lungs, but not in healthy skin^45^. Equivalent studies in patients with psoriasis are however, completely missing.

To address this gap, we performed a bulk-TCRSeq analysis of MR1-restricted MAIT cells sorted from peripheral blood of PV patients and healthy examinees. Our data demonstrate private MAIT TCRβ repertoire in both healthy and psoriatic examinees, with a few expanded clonal variants occupying a small part of the PV repertoire, mostly in older subjects. Moreover, we found evidence of preferential TRBV usage in CDR3β sequences from PV patients, and identified several MAIT TCRβ clonotypes that were shared between PV patients but remained undetected in the CDR3β collection of healthy controls. Overall, our data provide a first insight into the TCRβ repertoire of peripheral MAIT cells in PV and warrant further evaluation of its diagnostic and clinical significance.

## Results

### Subject characteristics

Baseline characteristics of the study participants are presented in Table 1. No significant differences in the studied properties were found between the PV and control participants. The PV patients exhibited varying degrees of disease severity (PASI score range: 4–30.2), with the majority having moderate to severe psoriasis and a longer history of disease. Most of the control and PV group participants were overweight and had a low systemic inflammatory burden. Furthermore, no significant differences were observed in the total frequencies or compartmental distribution of MAIT cells between the two groups, but there was an expected inverse correlation between age and the proportion of MAIT cells (ρ = − 0.54, P = 0.033). Positive history of CMV infection was evident in both groups, in line with previous reports of significant CMV prevalence in the Croatian population^46^.

**Table 1:** Study participants.

	PV (N = 10)	Controls (N = 10)	P
Gender (M/F)	8/2	8/2	1*
Age	34 (29.5–39)	34 (28.5–37)	0.591**
PASI	20.1 (5.35–23)	–	–
DLQI	10 (4.25–11)	–	–
Duration of disease (years)	11 (5.5–15.5)	–	–
BMI (kg/m^2^)	28.1 (27.4–29.6)	27.5 (26.5–29.2)	0.711**
CRP (mg/L)	0.74 (0.45–0.90)	1.4 (0.7–3.00)	0.222**
Anti-CMV IgG (pos/neg)	7/2	10/0	0.210*
Anti-CMV IgG (AU/mL)	84.9 (35.6–101)	100.8 (83.9–119.2)	0.141**
Leuk (× 10^9^/L)	6.7 (5.9–7.9)	5.7 (4.7–6.7)	0.031**
MR1-tet^+^ TCRVα7.2^+^ (%, of total CD3^+^)	3.34 (2.2–5.05)	3.64 (2.79–5.38)	0.637**

Continuous data are shown as median (interquartile range).

*BMI* body mass index, *CRP* C reactive protein, *CMV* cytomegalovirus, *DLQI* dermatology life quality index, *IgG* immunoglobulin G, *PASI* psoriasis area and severity index, *PV* psoriasis vulgaris.

*Fisher’s exact test, ** Mann-Whitney *U* test.

### Data overview

After correction for erroneous clonotypes, a total of 4.27 × 10^6^ TCR sequences were available for analysis, with an average of 2.67 × 10^5^ reads obtained per sample (Supplementary Table 4). The number of sequencing reads per repertoire varied between 3.43 × 10^4^ and 5.78 × 10^5^, while the number of unique clonotypes ranged from 1621 to 10438.

**Figure 1:**
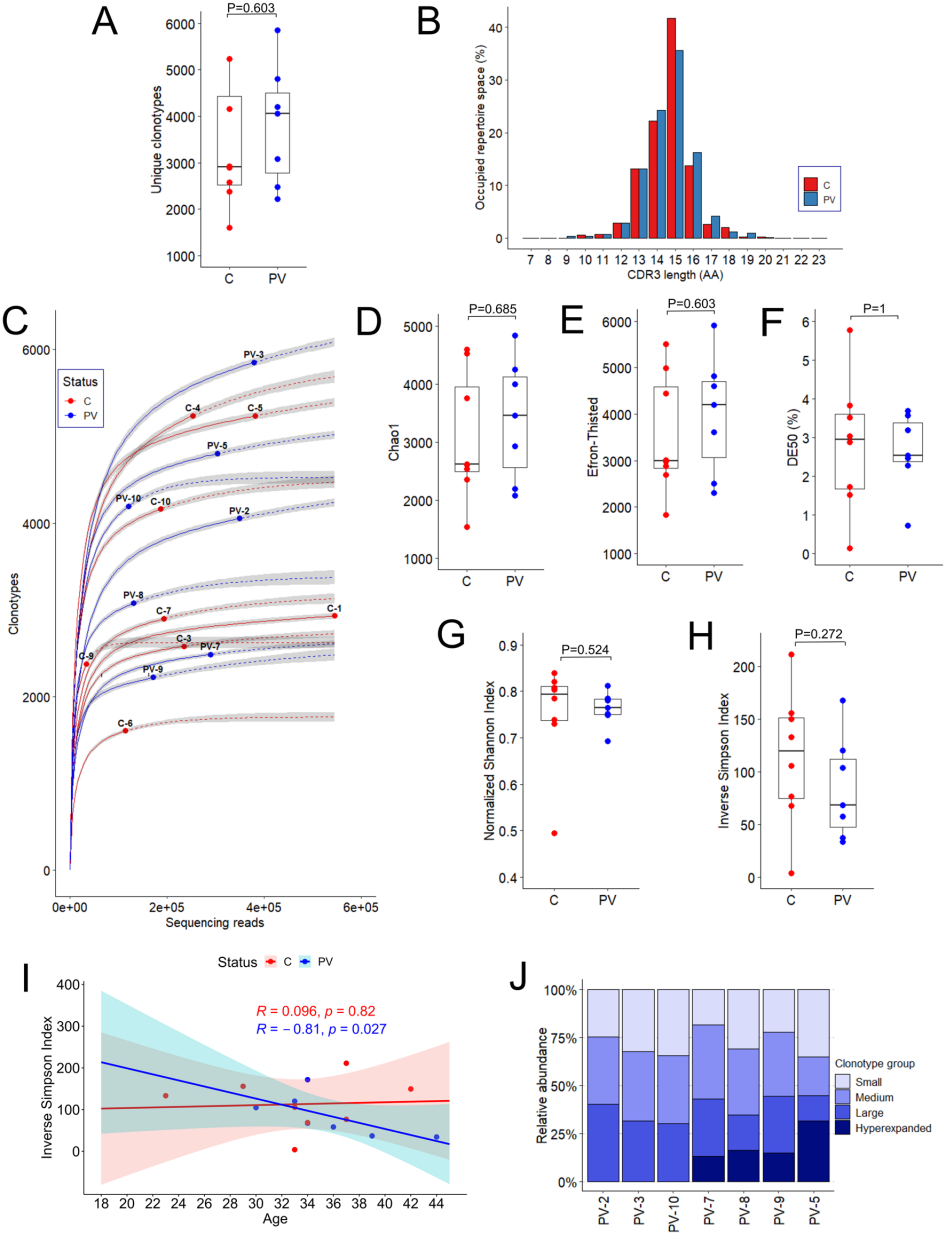
Baseline characteristics and diversity measures of the TCRβ repertoires of psoriasis patients and healthy individuals. (**A**) Number of unique clonotypes per sample. (**B**) The mean CDR3 amino acid length distribution within PV and control repertoires. (**C**) Rarefaction curves for each PV and control sample. The interpolated and extrapolated regions of the rarefaction curves are denoted by the solid and dashed lines, respectively, while the exact sample size and diversity are indicated by the dots. The shaded areas indicate 95% confidence intervals. (**D**) Chao1 diversity estimator. (**E**) Efron-Thisted diversity measure. (**F**) Distribution of DE50 values. (**G**) Normalized Shannon index values. (**H**) Inverse Simpson index values. (**I**) Correlation between age (years) and Inverse Simpson index within PV and control groups indicates the age-related diversity pattern in PV, Spearman’s rank correlation test. (**J**) Stacked barplots showing the relative frequency of clonotypes. Repertoires are ordered by patients’ increasing age on the X-axis. Categories of clonotype frequency in relative frequencies: “small” (< 0.0005), “medium” (0.0005–0.005), “large” (0.005–0.05) and “hyperexpanded” (0.05–1). The P-values in the boxplots were calculated with the Mann–Whitney test. Horizontal lines represent median with interquartile range.

The clonotype number in PV was not significantly different in comparison to healthy controls (Table 2, Fig. 1A).

**Table 2:** Descriptive characteristics of MAIT TCRβ clonotypes.

	PV	Controls	P*
Sequencing reads (N)	2.96 × 10^5^ (1.62 × 10^5^–3.57 × 10^5^)	2.14 × 10^5^ (1.69 × 10^5^–2.85 × 10^5^)	0.564
Clonotypes (N)	4125 (2929–5065)	2914 (2529–4430)	0.372
Clonotype frequency (%)	0.024 (0.019–0.034)	0.034 (0.023–0.039)	0.372
CDR3 length (nt)	43.96 (43.58–44.31)	44.03 (43.55–44.43)	0.875
Inserted nucleotides (N)	4.73 (4.61–4.93)	4.66 (4.52–4.93)	0.958
NDN size	12.34 (11.9–12.7)	12.17 (12.05–12.68)	0.793
Convergence	1.033 (1.028–1.036)	1.031 (1.028–1.033)	0.431

Data are shown as median with interquartile range.

*Mann–Whitney *U* test.

The length of the CDR3 region was highly variant (7–23), although most clonotypes (> 92%) contained CDR3 fragments of 12 to 16 amino acids in length (Fig. 1B). There was, however, no significant difference in CDR3 length distribution between PV and control groups (Table 2).

The junctional diversity, as judged by the number of nucleotides inserted into the V-D and D-J joints, and the number of nontemplated nucleotides (NDN), did not differ in the case-control comparison, nor did convergence, i.e. the number of unique CDR3 nucleotide sequences encoding the same CDR3 amino acid sequence (Table 2).

### The peripheral MAIT TCRβ repertoire of PV patients is highly diverse and expands with age

To provide a comprehensive measure of immune repertoire diversity, accounting for both repertoire richness and evenness, five different diversity indices (Chao1, Efron-Thisted, DE50, Normalized Shannon Index, and Inverse Simpson Index) were employed. The rarefaction plot was used in addition to visualize the relationship between sequencing depth and the estimated number of unique TCRβ clonotypes (Fig. 1C). One PV sample (female, 19 years of age) deviated from the rarefaction curves of the remaining sample collection and was thus excluded from diversity analysis.

The difference in lower bound diversity estimators Chao1 (Fig. 1D) and Efron-Thisted (Fig. 1E), which account for the number of singleton and doubleton clones in each sample, was not observed, thus providing evidence of similarly distributed low-frequency clones in both PV patients and healthy controls. High-frequent clonotypes, defined by the DE50 index as species occupying at least 50% of all sequencing reads, accounted for less than 3% (Fig. 1F), of all clonotypic variants in both groups, suggesting presence of only few expanded clonotypes in MAIT TCRβ repertoires of PV patients and healthy controls.

The normalized Shannon index (0–1 range), which measures both richness and evenness of clonal distribution by considering singleton frequency, was greater than 0.7 in both sample groups, indicating high clonotype abundance and versatility of MAIT TCRβ repertoire in PV and healthy examinees (Fig. 1G). When considering Simpson index as a measure of clonotype richness and evenness in clonotype distribution, similarly low values were noticed in both PV and control samples, confirming high abundance of equally distributed clonotypes (an inverse Simpson index was used in graphic presentation of data, Fig. 1H). Of interest, the Inverse Simpson Index declined with increasing age in PV, but not in control group (Fig. 1I), an effect which was dominantly driven by an increase in hyperexpanded clonotype compartment, a community of clonotypes comprising > 5% of the repertoire (Fig. 1J). This association was even more pronounced when data from one excluded, young female PV participant were incorporated in the analysis (Sperman’s ρ = − 0.874, P = 0.005). No evidence of confounding by sequence read numbers (P = 0.479, rank correlations against age) and disease duration (P = 0.818, rank correlations vs*.* age) was found, supporting the role of aging in TCRβ clonotype expansion. This well-established feature of MAIT and general T cell repertoires in healthy elderly people (> 60 years of age), was not expected in our younger subjects (median age 34 years). Age-related clonotype expansion was indeed absent in our healthy controls but was clearly noticeable in age-matched PV patients, indicating premature manifestation of age-related effects under PV conditions.

Overall, our results support significant MAIT TCRβ repertoire diversity, in both PV patients and healthy controls, featuring few expanded clonotypic variants next to the great number of highly diverse low frequency TCRβ clones. Moreover, increasing age was identified as an important confounder of peripheral MAIT TCRβ clonotype expansion under PV conditions. The early onset of MAIT TCRβ clonotype expansion in our middle-aged PV patients might be driven by PV-related systemic inflammation. To verify this hypothesis further longitudinal studies of MAIT TCRβ repertoire and function are necessary.

### The MAIT TCRβ repertoire of PV patients is skewed toward TRBV6-4/TRBJ2-3, TRBV20-1/TRBJ1-1 and TRBV15/TRBJ2-6 gene associations

**Figure 2:**
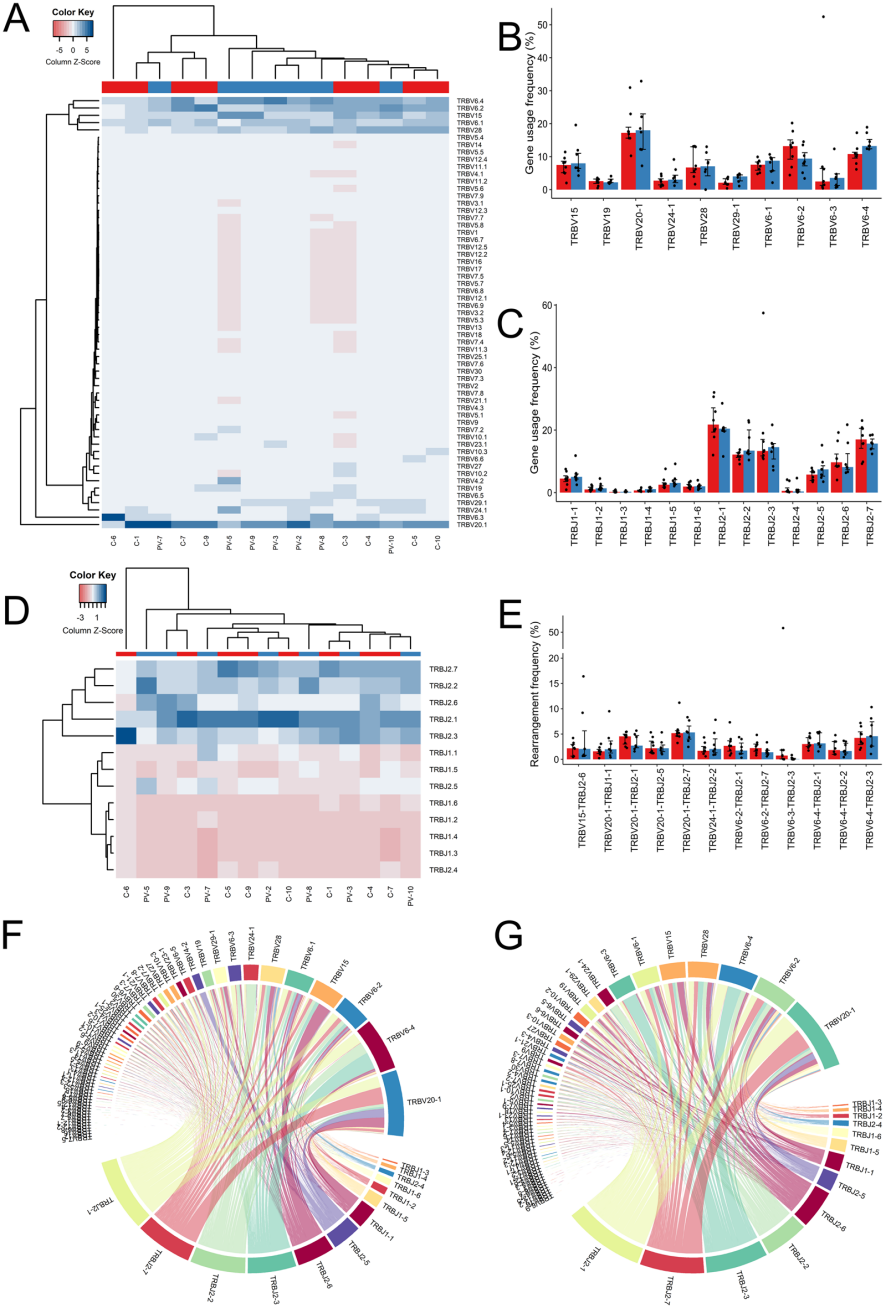
Analysis of TRBV/J gene usage and V-J pairings in MAIT TCRβ repertoires. (**A**) Hierarchical clustering of TRBV gene usage in PV and healthy control samples. (**B**) Relative usage frequencies of 10 most frequent TRBV gene variants for PV and healthy samples. (**C**) Relative usage frequencies of TRBJ variants for PV and healthy samples. (**D**) Hierarchical clustering of TRBJ gene usage. (**E**) Relative rearrangement TRBV/TRBJ frequency for PV and healthy samples. (**F**) Circos plot of V-J recombination events in the PV and (**G**) control groups (**G**). In (**B**,**C**,**E**) the bar height indicates the median frequency, error bars show IQR. P values were calculated by the Mann–Whitney test and corrected for multiple testing with the Benjamini–Hochberg method. None of the p values reached statistical significance (p < 0.05) and were thus not presented in graphs. In (**A**,**D**) Sequencing data were clustered based on Euclidean distance between TRBV/TRBJ segment usage, calculated according to default settings of the VDJTools *CalcSegmentUsage* function. Z-scores represent the relative frequency of each gene segment. Dendrograms show hierarchical clustering of samples and gene segments.

The TRBV/J gene usage was investigated in the next step. The resulting hierarchical clustering, performed using Euclidean distance, revealed highly diverse TRBV/J gene usage, with no discernible patterns between PV and healthy controls (Fig. 2A,D). Specifically, 59 TRBV gene variants were detected across the whole sample collection (Fig. 2A), 38 of which were present in all annotated TCRβ sequences. As anticipated, MAIT TRBV gene usage was skewed toward TRBV20-1 and TRBV6 gene family members, accounting for > 50% of all TRBV variants within the MAIT TCRβ repertoire. The TRBV15, TRBV28, TRBV24-1, TRBV29-1, and TRBV19 were among the 10 most frequently used genes, which, along with TRBV20-1 and TRBV6, comprised nearly 80% of all TRB sequences (Fig. 2B). Compared to healthy controls, the MAIT TCRβ repertoire of PV patients was nominally enriched in TRBV11-2 and TRBV6-4 genetic variants but following post-hoc correction for multiple testing (Benjamini-Hochberg method) the significance was lost, likely reflecting the limitations of our small sample size (data not shown). Of interest, preferential TRBV6-4 usage was previously described in adult blood Tet-MR1^+^MAIT cells of healthy donors^47^ supporting the need for further gene usage assessment in larger PV cohort.

TRBJ gene usage analysis revealed 13 different TRBJ variants in the MAIT TCRβ chain composition (Fig. 2C,D), of which TRBJ2-1 gene accounted for >20% of all constituent TRBJ variants. Less frequent representatives of TRBJ2 family were TRBJ2-7, TRBJ2-2, TRBJ2-3, TRBJ2-6 and TRBJ2-5, whereas the remaining TRBJ1 family members comprised <16% of MAIT TRBJ repertoire (Fig. 2C). The frequency of TRBJ gene usage in MAIT TCRβ chain composition did not, however, differ between cases and controls.

The assessment of V-J recombination events involving 59 TRBV and 13 TRBJ gene segments revealed 614 and 605 distinct V-J pairings in the PV and control groups, respectively (Fig. 2F,G, Supplementary Table S5). The composition of the most frequent TCRBV-J pairings was similar between the PV and control groups, as evidenced by the presence of TRBV20-1-TRBJ2-1/2-5/2-7, TRBV6-4-TRBJ2-3/2-1, TRBV15-TRBJ2-6, TRBV24-1/TRBV2-2, and TRBV6-2-TRBJ2-1 associations, comprising >25% of all TCRβ sequences in both groups. Nonetheless, several distinct TRBV/TRBJ associations displayed varying frequencies in each group. In particular, the TRBV6-4/TRBJ2-3 combination had the highest prevalence in the PV cohort, accounting for 5.49% of CDR3β sequences, whereas TRBV20-1-TRBJ2-7 emerged as the most abundant in healthy MAIT TCRβ repertoires, comprising 6.66%. Their respective counterparts in healthy and PV repertoires occupied 4.32% and 5.37% of TCRβ collection, with no significant difference in case-control comparison (Fig. 2E). The representation of TRBV6-4-TRBJ2-2, TRBV20-1-TRBJ1-1 and TRBV15-TRBJ2-6 combinations was in addition, more common in the MAIT TCRβ clonotypes of PV patients (Fig. 2F), while the TRBV6-3-TRBJ2-3 and TRBV6-2-TRBJ2-7 gene associations were more prevalent in the MAIT TCRβ repertoires of healthy subjects (Fig. 2G), but none were significantly different in healthy vs. PV repertoire comparison (Fig. 2E).

Collectively, our findings demonstrate a wide range of TRBV/J gene combinations in MAIT CDR3 rearrangements, confirming the preferential use of TRBV20-1, TRBV6 and TRBJ2 family gene members in both sampled groups, with no significant case-control differences in TRBV/TRBJ gene usage and prevalence of the most common V-J pairs.

### The public repertoire of circulating MAIT cells comprises unique clonotypes corresponding to published blood and skin TCRβ collections of PV patients

**Figure 3:**
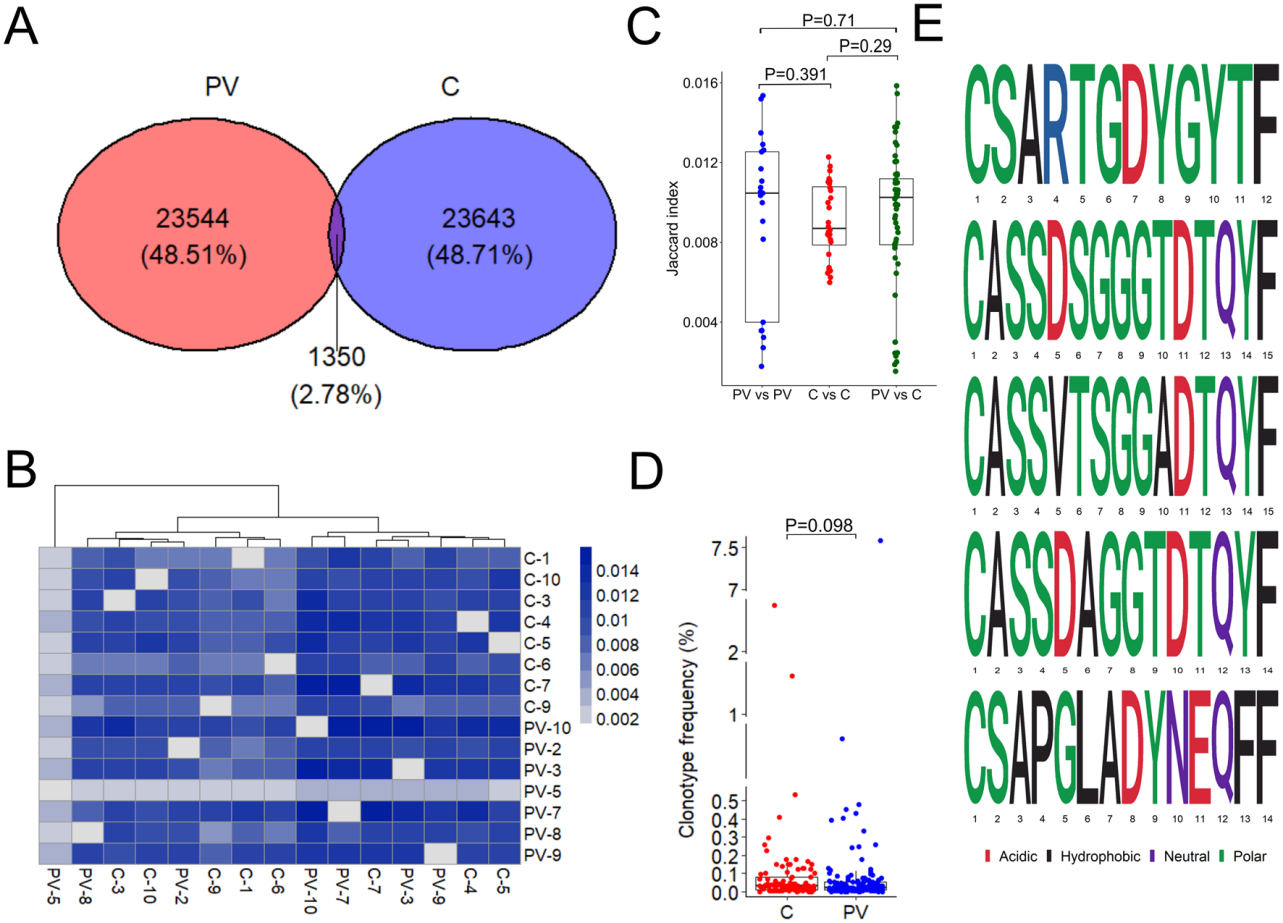
Analysis of clonotype overlap, public clonotypes, and psoriasis-specific clonotypes. (**A**) Venn diagram depicting overlap of clonotypes between pooled PV and control repertoires. (**B**) Heatmap of the Jaccard index indicating higher (dark blue) or lower (light blue) overlap between two sample sets. (**C**) Jaccard index values within PV, within control, and between PV and healthy group. (**D**) Frequencies of top 11 public clonotypes across control and PV samples. P-values were calculated by Mann–Whitney test. (**E**) Amino acid sequences of five clonotypes frequently shared between PV patients (≥ 4/7) but absent in healthy repertoires.

In the next step, the assessment of public and private clonotype repertoire was performed. The initial inspection of the peripheral MAIT TCRβ repertoires in all 15 participants revealed 48537 unique clonotypes, the 24894 of which were observed in PV samples, while 24993 belonged to the healthy TCRβ transcript pool. Interestingly, 97.2% of all clonotypes were private, i.e. unique to the individual repertoires, while only a small proportion (2.78%) was shared between at least one PV and one healthy control sample (Fig. 3A). This matches well the observations by Garner et al., reporting that MAIT cells show surprising clonal diversity, with TCR repertoires shared across tissues but not between individuals^18^. Among the public clonotypic variants, 11 sequences stood out as being present in the majority (at least 11 out of 15) of the examined MAIT TCRβ repertoires (Table 3A). All these sequences matched MAIT-affiliated CDR3β sequences enlisted in the recently published unconventional TCR database (UcTCRdb^48^, Supplementary Table S6), supporting the influence of the common antigen(s) that shape the MAIT TCRβ repertoire, regardless of the case-control status. Six of those were concordant with MAIT TCRβ sequences of donors having asymptomatic Mycobacterium tuberculosis (Mtb) infection, even though our sample cohort was tested against active Mtb disease. Of interest, three of six putatively Mtb-related clonotypes were also affiliated with CMV epitopes enlisted in the VDJdb database^49^. Their confidence score was however equal to zero (10x dextramer sort method), suggesting false VDJdb antigen annotation, or alternative reactivity depending upon different TCRα chain specificity. These uncertainties illustrate the limits of the *in silico* approach to MAIT TCRβ antigen specificity.

**Table 3:** Public MAIT clonotypes with the highest incidence in peripheral blood: (A) from all participants; (B) from PV patients; (C) from control subjects.

Clonotype (aa)	TRBV segment	TRBJ segment	PV occurrence	Control occurrence	Total occurrence	Public frequency (%)
(A)						
CASSYRDTGELFF	TRBV6-2	TRBJ2-2	5/7	8/8	13/15	17.98
CASSDRDTGELFF	TRBV6-4	TRBJ2-2	6/7	6/8	12/15	20.42
CASSDSSGANVLTF	TRBV28	TRBJ2-6	6/7	6/8	12/15	7.45
CASSELAGGPDTQYF	TRBV6-1	TRBJ2-3	6/7	6/8	12/15	6.97
CASSDSNTGELFF	TRBV6-4	TRBJ2-2	6/7	6/8	12/15	6.32
CSARLAGGQETQYF	TRBV20-1	TRBJ2-5	6/7	6/8	12/15	5.59
CASSDSSGSTDTQYF	TRBV6-4	TRBJ2-3	6/7	5/8	11/15	3.42
CASSGTSGSTDTQYF	TRBV6-4	TRBJ2-3	5/7	6/8	11/15	3.14
CASSETSGSTDTQYF	TRBV6-4	TRBJ2-3	6/7	5/8	11/15	2.96
CSARQGDTEAFF	TRBV20-1	TRBJ1-1	5/7	6/8	11/15	1.72
CASSDSDTGELFF	TRBV6-4	TRBJ2-2	4/7	7/8	11/15	1.65
(B)						
CASSDSGGSYNEQFF	TRBV6-4	TRBJ2-1	6/7	2/8	8/15	0.19
CASSEAGGTDTQYF	TRBV6-4	TRBJ2-3	6/7	2/8	8/15	0.18
CASSDSSGSYNEQFF	TRBV6-4	TRBJ2-1	6/7	3/8	9/15	0.48
CASSELAGGQETQYF	TRBV6-1	TRBJ2-5	6/7	3/8	9/15	0.38
CASSYGTSGYEQYF	TRBV6-2	TRBJ2-7	6/7	4/8	10/15	1.41
CSARTGDTEAFF	TRBV20-1	TRBJ1-1	6/7	4/8	10/15	0.82
CSARTGDYGYTF	TRBV20-1	TRBJ1-2	5/7	0/8	5/15	0.01
CASSAGDTGELFF	TRBV6-4	TRBJ2-2	5/7	2/8	7/15	0.06
CASSPTENTEAFF	TRBV6-4	TRBJ1-1	5/7	2/8	7/15	0.05
CASSDSTSGSNEQFF	TRBV6-4	TRBJ2-1	5/7	3/8	8/15	0.36
CASSDRGTGELFF	TRBV6-4	TRBJ2-2	5/7	3/8	8/15	0.15
CASSEGSNQPQHF	TRBV6-4	TRBJ1-5	5/7	3/8	8/15	0.1
CASSDSSTDTQYF	TRBV6-4	TRBJ2-3	5/7	4/8	9/15	0.57
CASSDTSGSTDTQYF	TRBV6-4	TRBJ2-3	5/7	4/8	9/15	0.54
CASSYSTSGGNEQFF	TRBV6-2	TRBJ2-1	5/7	4/8	9/15	0.37
CASSDNSGANVLTF	TRBV6-4	TRBJ2-6	5/7	5/8	10/15	1.21
CSARGDRDYEQYF	TRBV20-1	TRBJ2-7	5/7	5/8	10/15	0.75
(C)						
CATSRDSSGANVLTF	TRBV15	TRBJ2-6	2/7	7/8	9/15	0.97
CASSYSDTGELFF	TRBV6-2	TRBJ2-2	4/7	6/8	10/15	1.02
CASSYNSGANVLTF	TRBV6-2	TRBJ2-6	3/7	6/8	9/15	0.79
CASSYGTSTDTQYF	TRBV6-2	TRBJ2-3	3/7	6/8	9/15	0.69
CASSELAGGYNEQFF	TRBV6-1	TRBJ2-1	3/7	6/8	9/15	0.58
CASSELAGGTDTQYF	TRBV6-4	TRBJ2-3	3/7	6/8	9/15	0.31

Overlapping clonotypes public in both the PV/control group and all samples (Table A) have been omitted from Table B and Table C for clarity. Public frequency (%) was calculated as the percentage that each clonotype occupies in a sample of pooled public clonotypes. Occurrence signifies the number of samples within the observed group in which the clonotype appears.

A higher number of concordant clonotypes was nevertheless detected when the complete collection of public clonotypes was considered in comparison to previously published TCRβ repertoires of cells from peripheral blood^50,51^, lesional^39,42,50^ and non-lesional^38^ skin of PV patients and healthy controls (Supplementary Table S6). The highest number of public CDR3β sequences that matched our MAIT TCRβ collection was observed within the repertoire of bulk peripheral T cells of PV patients (Supplementary Table S6), encompassing blood and tissue-tropic T cells, the latter of which expressed a skin-homing cutaneous lymphocyte-associated antigen (CLA). In addition, a number of matching clonotypes was seen across the healthy, non-lesional and lesional skin TCRβ repertoire comparisons, respectively, suggesting a link between MAIT TCRβ abundance and disease progression.

The analysis of public repertoire in PV patients revealed a total of 710 (2.85%) public clonotypes, 27 of which were shared between at least 5 out of 7 analyzed PV libraries (Table 3B). The public repertoire of healthy examinees contained 867 (3.47%) public clonotypes, including 15 most frequently shared variants presented in Table 3C. Those public MAIT TCRβ sequences of PV and healthy subjects did not dominate within the corresponding individual repertoires but occupied a comparatively similar middle frequency clonotype compartment (PV vs. C, median (IQR): 0.024 (0.009–0.053) vs. 0.033 (0.012–0.079 P = 0.098, Fig. 3D). The clonotype overlap was also assessed by using Jaccard index (JI), defined as the ratio of the public vs. total clonotype number in both sample groups. The obtained JI values ranged between 0.0015 and 0.0158, without significant difference in cross-group (PV vs PV, C vs C, median (IQR): 0.0105 (0.0039–0.0125), 0.0087 (0.0079–0.0108)) or case-control (PV vs C, median (IQR): 0.0103 (0.0079–0.0112) comparisons (Fig. 3C).

Of interest, more than half of the most abundant public clonotypes of healthy controls were also present in PV repertoires, whereas highly shared PV clones were less frequently observed in the control group. One notable example is the CSARTGDYGYTF clonotype, which was detected in 5 out of 7 PV patients but was completely absent from healthy repertoires. Certain control exclusive clonotypes, such as the commonly observed CSAREGDTEAFF variant which was present in 5 out of 8 healthy control samples, were identified as well. The subsequent PV vs. healthy comparison of public MAIT TCRβ clones revealed a total of 420 healthy- and 336 PV-unique clonotypes that were shared within, but not between groups, with eight being frequently shared (≥ 4/7) in healthy, and five in PV. Commonly shared PV unique variants, namely CSARTGDYGYTF, CASSDSGGGTDTQYF, CASSVTSGGADTQYF, CASSDAGGTDTQYF and CSAPGLADYNEQFF, had different CDR3 loop motifs, characterized by distinct TRBV and TRBJ gene usage and length variability (Table 4, Fig. 3E). The median frequencies of these clonotypes were relatively low, with the most abundant one, CSAPGLADYNEQFF, occupying 1.6% of the individual TCRβ repertoire (PV-7). The CSARTGDYGYTF clonotype, another member of our PV-exclusive clonotype pool, has been recently reported as part of the peripheral non-skin homing CLA^-^ T cell repertoire of psoriatic patients^50^. Two additional clonotypes that were unique to PV, the CASSDSGGGTDTQYF and CASSDAGGTDTQYF, were previously described in lesional^38,39,50,51^ and non-lesional^38^ psoriatic skin, supporting recent evidence of significant repertoire overlap, and potentially, recirculation between lesional skin and blood T cells^50^. Such T cell communities have been characterized as highly polyclonal, with a potential to invade non-skin body sites and thereby induce systemic inflammation^50^. Both clonotypes were however, also matched with Mtb-activated CD8^+^CD69^+^CD154^+^TRAV1-2^+^, and *Salmonella* *Paratyphi A* challenged peripheral blood TCR repertoires of healthy donors (presented in Supplementary Table S6), supporting their alternative roles that go beyond PV.

**Table 4:** Clonotypes found in more than 50% of PV patients, but not among healthy subjects.

Clonotype (aa)	TRBV	TRBJ	Occurences (PV)	Frequency in pooled PV clonotypes (%)
CSARTGDYGYTF	TRBV20-1	TRBJ1-2	5/7	0.25
CASSDSGGGTDTQYF	TRBV6-4	TRBJ2-3	4/7	0.07
CASSVTSGGADTQYF	TRBV6-1	TRBJ2-3	4/7	0.05
CSAPGLADYNEQFF	TRBV29-1/TRBV20-1	TRBJ2-1	4/7	0.15
CASSDAGGTDTQYF	TRBV6-4	TRBJ2-3	4/7	0.11

To test whether the five PV-specific clonotypes could be linked to antigen specificity in PV, the probability of generation model was employed using VDJRec pipeline^52^. Results suggested that these clonotypes may be commonly shared due to a higher probability of generation through convergent recombination rather than specific antigen selection in PV. Moreover, to analyze whether public clonotypes were statistically increased or decreased in PV, one-tailed Fisher’s exact test (Benjamini-Hochberg correction) was applied. The results did not reveal any significance, likely due to the small sample size.

Nonetheless, considering the relatively conserved antigen-recognition patterns of MAIT cells and the potential influence of common microbial triggers in psoriasis^23,34,53–55^, the true significance of these clonotypes in PV warrants further validation in larger sample sizes and paired skin samples.

## Discussion

MAIT lymphocytes are innate-like T cells that make up a considerable part of blood and epithelial tissue immune community, poised for rapid, anti-microbial effector functions at barrier sites. As such, MAIT cells have been implicated in the pathogenesis of psoriasis vulgaris, but the TCR repertoire, which underlies their reactivity against yet unknown antigens in psoriasis, is largely unexplored. Given the conserved nature of the MAIT TCRα chain, the repertoire of TCRβ clones carries greater potential for the identification of TCR variants that could contribute to the occurrence and/or recurrence of psoriasis. Therefore, we used bulk-TCRSeq to estimate TCRβ repertoire composition and its diversity in flow-sorted, peripheral blood MR1^+^ TCRVα7.2^+^ MAIT cells from clinically well-characterized PV patients and healthy controls.

We found evidence of highly private and diverse MAIT TCRβ repertoires, largely unique to examined individuals, irrespective of their differences in case-control status. Few expanded clonotypes accounted for the majority of MAIT CDR3β collection in both groups, supporting high TCRβ oligoclonality, previously demonstrated for CD161^+^TCRVα7.2^+^ MAIT and blood T cells of healthy and affected individuals, respectively. Our findings correlated well with a recent single-cell TCRSeq analysis of MR1-restricted, blood MAIT cells of healthy individuals, corroborating high TCRβ clonotype diversity^18,20^, donor-unique repertoire, small public clonotype content, and high CDR3β amino acid length variability. These features were equally representative of the MAIT TCRβ repertoire of our PV subjects, with few relevant distinctions, including, high occurrence of TRBV6-4-TRBJ2-2, TRBV20-1-TRBJ1-1 and TRBV15-TRBJ2-6 rearrangements, and existence of public variants shared among PV, but not healthy MAIT repertoires. The expansion of most abundant clonotype compartment with age, was an additional feature of MAIT TCRβ repertoire, unique to older PV subjects, and reminiscent of large clonal expansions of MAIT TCRβ repertoire which usually take place in older healthy adults^56^. Innate cell immunosenescence that occurs under conditions of chronic inflammation in PV potentially contributes to the observed phenomenon^57^.

Skewed TCRβ repertoire, marked by increased usage of TRBV6 and TRAV20 gene segments, has been previously affiliated with TCRβ clones of the putative pathogenic T cells isolated from clinically resolved lesional and non-lesional psoriatic skin^39^. One of our PV-unique public clonotypes (CASSQDLAGGPDTQYF), matched the CDR3β sequences of these putative culprit cells, while more frequent PV-associated variant, CSARTGDYGYTF, coincided with the peripheral, non-skin homing T cell repertoire of psoriatic patients^50^. Two additional, PV-specific variants (CASSDSGGGTDTQYF and CASSDAGGTDTQYF), were matched by multiple TCRβ libraries from lesional^38,50,51^ and non-lesional^38^ skin samples of PV patients, indicating their potential relevance and close relationship with cutaneous T cell clones. In addition, a number of concordant clonotypes (9/16) was found by comparing the most frequent, public MAIT TCRβ variants from our PV cohort, and the published record of CDR3β sequences of skin-tropic blood T cells, lesional and non-lesional tissues of PV patients. These concordances support the findings of a recent comparative analysis of peripheral and cutaneous T cell repertoires in PV^50^, demonstrating that clonally expanded T cells from lesional skin can be detected within the circulation. Accordingly, a confirmatory study in paired blood and skin samples is warranted.

Public clonal sequences accounted, however, for a small proportion of the examined MAIT CDR3β collections, with PV-associated public variants modestly represented within individual MAIT repertoires. The remaining part of the public repertoire was shared between PV and healthy subjects, and fully aligned with CDR3β sequences included in the UcTCRdb database^48^, indicating the influence of common antigens in the construction of the peripheral MAIT TCRβ repertoire. The source of these antigens, however, remains unknown, together with the tissue in which these MAIT cells are expanded and educated. Judging by the number of public clonotypes matching the repertoire of Mtb-infected patients, common ribityl commensals could be an important driving force behind the shape of the peripheral MAIT repertoire in both PV patients and healthy controls. Presence of alternative riboflavin-producing bacteria, including PV-related gut^53^ and skin^54,55,58^ commensals, is possibly also reflected in our public MAIT repertoire. Moreover, matching clonotypes revealed between our PV-exclusive public collection and non-skin homing CLA^-^ repertoire of psoriatic patients, potentially mirror T cell species sensitized against non-skin specific antigens^59,60^, that may facilitate development of common psoriatic comorbidities and systemic inflammation. Private sequences detected within individual MAIT repertoires require further investigation as well. The pathogenic relevance of PV-exclusive public sequences, found here, should be furthermore, validated in larger number of subjects, using paired samples of blood and skin. Limitations imposed by current study settings, particularly the unpaired nature of our TCRβ data obtained through relatively short read sequencing strategy, hindered, an exhaustive, pairwise analysis of complete MAIT TCR repertoire, making predictions and conclusions about its antigenic specificity and potential pathogenicity very difficult and uncertain. Future estimates of MAIT TCRβ repertoire in PV would no doubt also benefit from methodological upgrades in terms of single-cell TCR sequencing and targeted MAIT cell panel, particularly unconventional population with varying degree of MR1-tetramer and TCRVα7.2 receptor expression, which have been recently implicated in PV^61^.

Despite these shortcomings, our data shed light on hitherto unexplored parts of the TCR repertoire of MR1-restricted MAIT cells in psoriasis, revealing their extremely diverse, individually unique repertoire, which significantly expands with age. New evidence also reveals preferential use of specific TRBV/TRBJ gene combinations in peripheral MAIT cells of PV subjects, and confirms the existence of several public, PV-unique clonotypes that reflect parts of the peripheral and lesional inflammatory repertoire. Identified TCRβ clones require further validation in paired skin samples of PV patients and may prove as a promising diagnostic or therapeutic target.

## Methods

### Patient recruitment and sample collection

This study included 10 clinically active psoriasis vulgaris (PV) patients and 10 healthy adults. Affected individuals were recruited according to the clinical and pathohistological findings of skin biopsy samples, collected during standard diagnostic procedures at the Department of Dermatology and Venereology, Clinical Hospital Centre Osijek. Disease severity and impact on quality of life were assessed via psoriasis area severity index (PASI) and dermatology life quality index (DLQI). The control group consisted of healthy adults, age- and gender-matched to the patient group, which were selected during regular dermatologic care of benign, non-infectious, and non-allergic skin changes. Patients on systemic immunomodulatory, cytostatic, photochemotherapy (psoralen, UVA, radiation) or phototherapy (narrow-spectrum UVB), with associated autoimmune, malignant, and infectious diseases or allergic reactions within six weeks before diagnostic processing were excluded from the study. Demographic data and anthropometric measurements were collected at the time of the recruitment, and serologic markers of bacterial (QuantiFERON-TB Gold test) and viral exposure (anti-CMV IgG, anti-CMV IgM, anti-HBsAg, anti-HCV) were probed in all participants. Complete blood count (CBC), C-reactive protein (CRP) serum levels, and erythrocyte sedimentation rate (ESR) were assessed as well. An informed consent was signed by all participants prior to the sample collection. The study was approved by the Ethics Committee of the Faculty of Medicine in Osijek (Certificate No. 2158-61-07-19-126, October 11, 2019) and the Ethics Committee of the Clinical Hospital Centre Osijek (Certificate No. R2-12487/2019, September 12, 2019). All experiments were performed in accordance with relevant guidelines and regulations. Demographic and clinical characteristics of the recruited subjects are summarized in Table 1.

### Peripheral blood mononuclear cell (PBMC) harvesting and cryopreservation

Peripheral blood samples (20 ml) were collected in heparin-treated tubes and immediately processed for PBMC separation in Lymphoprep gradient density medium (Stemcell Technologies, Vancouver, Canada). In short, whole blood was diluted 1:1 with saline (0.9% (w/v) NaCl), layered onto Lymphoprep, and centrifuged for 25 minutes at 800 × g without brake. The milky layer of PBMCs was harvested, washed (1x PBS) and pelleted (550 × g for 10 min) in two consecutive steps. Collected PBMCs were counted (LUNA-II Automated Cell Counter, Logos Biosystems), pelleted and cryopreserved in 3 × 10^6^ cell aliquots suspended in 0.5 ml of cold FBS (Biosera, France), and an equal volume of pre-chilled (4°C) freezing medium [FBS + 20% of DMSO (AppliChem)] added dropwise. Cryovials were placed in a Mr. Frosty container (Nalgene) at − 80°C for 48 h, before being transferred into a liquid nitrogen tank for long-term storage. Prior to cell sorting, cryopreserved PBMCs were thawed rapidly in a 37°C water bath and dropwise diluted with 10 mL of pre-warmed supplemented RPMI-1640 medium (10% FBS, 1% sodium pyruvate, 0.01M HEPES, Sigma-Aldrich). Thawed cells were pelleted by centrifugation at 350 x g for 8 minutes, and the cell sediment was resuspended in 5 mL of MACS buffer (1x PBS + 0.075% EDTA + 0.05% BSA), after which the number and cell viability were determined on a LUNA-II Automated Cell Counter.

### Flow cell cytometry staining, sorting and RNA extraction

On average, 13.3 × 10^6^ (min-max range: 6.84 × 10^6^–20.2 × 10^6^) thawed PBMCs were initially incubated with 5% human FcR blocking agent (TruStain FcX, Biolegend) for 10 minutes, before being stained in two successive steps of twenty minutes each; firstly with the MR1-5-OP-RU (5-(2-oxopropylideneamino)-6-D-ribitylaminouracil, 1:100, NIH Tetramer Core Facility^62^) conjugated tetramers, and then with a mixture of monoclonal antibodies against CD3ϵ (FITC, 1:250, clone UCHT1 gamma, prepared in the Department of Immunology and Biotechnology, University of Pécs, Hungary) and TCRVα7.2 (PE, 1:100, clone 3C10, BioLegend). After staining, cells were rinsed twice in MACS buffer, and prior to the S3e cell sorter (Bio-Rad Laboratories) acquisition, filtered through a 35 µm mesh to remove cell aggregates and debris. A portion of each sample (5 × 10^5^ cells) was stained with Live/Dead Fixable Near IR Dead fluorescent dye (ThermoFisher Scientific, USA) prior to antibody staining [CD3ϵ FITC (1:250, clone UCHT1 gamma, CD4 PE-Cy7 (1:200, clone SK3, eBiosciences), CD8a PerCP-Cy5.5 (1:200, clone RPA-T8, eBiosciences), TCRVα7.2 PE (1:100, clone 3C10, BioLegend) MR1-5-OP-RU conjugated tetramer [1:100, NIH Tetramer Core Facility] and analysed by flow cytometry (FACS Canto II, BD). Cells were live gated according to live/dead staining and then analyzed for combinations of antigen expression to determine PBMC composition. On average a minimum of 30,000 MR1-reactive T cells (min-max range: 30,000–270,000) were sort-purified form each PBMC sample (Supplementary Table S1) directly into TRIzol reagent (Sigma Aldrich) and used immediately for total RNA extraction according to the Direct-zol RNA MicroPrep Kit (Zymo Research, USA) instructions. Quantity and purity of obtained RNA were assessed by the QFX Fluorometer (Envoi, USA) using Qubit RNA High Sensitivity Assay kit. Extracted RNA samples were stored at − 80 °C until further analysis.

To verify cell purity, 5 randomly drawn blood samples were processed into PBMC aliquots and MR1-reactive cells were sorted into 100% FBS and assessed by post-sorting flow cytometry (DxFLEX, Beckman Coulter), confirming high cell homogeneity/purity (93.58 ± 6.53%) of the sorted MAIT cell population (Supplementary Fig. S1).

### TCRβ sequencing library preparation

Sequencing libraries were prepared with the AmpliSeq for Illumina Immune Repertoire Plus, TCR beta Panel kit, according to the protocol outlined in the manufacturer’s Reference Guide. In line, 10 ng of total RNA was first reverse transcribed (AmpliSeq cDNA Synthesis kit), and the subsequent amplification of TRB transcripts achieved by the 23 repetitive cycles of multiplex PCR. Primer dimers and partial amplicons were removed in the next three successive incubations with FuPa reagent (at 50 and 55 for 10 min, 62 for 20 min), and each library was further extended by the unique dual index (UDI) adapter ligation (at 22 for 30 min, 68 and 72 for 5 min). The adapter residues were removed by magnetic library purification using magnetic beads (MagSi-NGSPREP Plus, MagnaMedics Diagnostics B.V., Netherlands) and a 96-well magnetic stand. Refined libraries were amplified in the second amplification step (7 cycles at 98 °C for 15 s and 64 °C for 1 min), and further purified by two rounds of magnetic beads clean up to remove high-molecular-weight cDNA molecules and residual primers. The library size was checked by the 1.5% electrophoresis, revealing fragments with an average size of 300 bp. Library quantification was performed with the use of KAPA Library Quantification Kit and the QuantStudio 5 real-time PCR instrument (Thermo Fisher Scientific, USA). All qPCR reactions were run in triplicate, and QuantStudio Design & Analysis Software v 1.5.1 was used for data analysis. The following equation: $molarity \left(pM\right)=conc \left[qPCR (nM)\right]\times \frac{452\, bp}{average\, library\, size\, \left(bp\right)}\times 10000 (dilution)$, was applied for molarity calculations, where $conc \left[qPCR (nM)\right]$) presented the mean value of triplicate library measurements, and the average library size, the size of the library fragments obtained by gel electrophoresis. The sequencing libraries and PhiX control were next denatured and diluted according to the MiniSeq System Denature and Dilute Libraries Guide (v09), with the Standard normalization workflow used in library preparation. Finally, 5 µl of the denatured Phix control was mixed with 495 µL of pooled library sample, yielding a 1% PhiX spike-in control. High-throughput paired-end sequencing (2 × 150 bp read length, MiniSeq System High-Output Kit) was performed on the MiniSeq Illumina platform with FASTQ file generation, demultiplexing, and adapter-trimming options enabled.

### Sequencing run quality control and pre-processing of TCRSeq data

Sequencing run quality metrics were assessed using Illumina Sequencing Analysis Viewer software (version 2.4.7.) and the results are presented in Supplementary Table S2. To ensure the accuracy and robustness of the analysis, one out of the four excluded samples was withheld from further analysis due to low sequencing depth (2236 < 30,000 reads), and the remaining three were removed due to the anomalous sequencing output that exceeded the upper outlier read count threshold (812670, calculated as 75th percentile + 1.5 × IQR). The quality of the remaining paired-end data was assessed using FastQC tool (version 0.12.0, 45), and all samples were found to be of sufficient quality.

TCRSeq data in FASTQ format were pre-processed with MiXCR^63^ for read alignment and clonotype assembly. MiXCR identifies T-cell receptor sequences by combining the identification of V(D)J gene segments and CDR3 sequence reconstruction, accounting for PCR and sequencing errors. The TCR clonotype is defined according to unique nucleotide sequence. The output of MiXCR includes comprehensive information on the clonotype frequency, V(D)J gene segment usage, CDR3 nucleotide and amino acid sequence, enabling downstream analyses of TCR repertoire. A summary of the MiXCR assembly report is provided in Supplementary Table S3.

To account for residual erroneous clonotype variants, the *Correct* function of VDJTools^64^ was used to merge low-abundance with high-abundance clones of similar nucleotide sequence, reducing the total MiXCR clonotype output by less than 2% (Supplementary Table S4). The tables with the corrected clonotype frequencies were used for downstream analyses performed in VDJTools and immunarch^65^.

### TCRbeta repertoire analysis

Three bioinformatics tools, i.e. MiXCR (v 3.0.13.), VDJTools (v 1.2.1.), and immunarch (v 0.9.0) were employed for clonotype profiling, statistical analysis, and results presentation. To assess basic statistics, CDR3 length distribution, diversity indices, rarefaction curves, TRBV/TRBJ gene usage, TRBV-TRBJ pairing, and public clonotypes, we used the following VDJTools functions - *CalcBasicStats*, *CalcSpectratype*, *CalcDiversityStats*, *RarefactionPlot, CalcSegmentUsage*, *PlotFancyVJUsage*, and *JoinSample*, respectively. The *ApplySampleAsFilter* VDJTools routine was used in addition, to extract CDR3β sequences that matched with CDR3β variants of previously published TCRβ collections. Matching clonotypes, shared between at least 2 of the 15 samples tested, were identified after applying the *JoinClonotypes* routine. The outputs of these functions were visualized by VDJTools source-code R scripts that were modified only for aesthetic purposes. The *tidyverse*, *ggpubr*, *RColorBrewer*, *gplots* (*heatmap.2* function) and *VennDiagram* R packages were used in addition for generation of TRBV/TRBJ gene usage heatmaps and Venn diagram, respectively. Moreover, *geneUsage*, *getKmers*, and *repOverlap* immunarch functions were employed to generate bar graphs, position probability matrix, and Jaccard index overlap.

### Statistical analysis

Statistical analysis was conducted using R software (v.4.1.1). Descriptive statistics, including measures of central tendency and dispersion were used to summarize the data (further details are available as legends to the figures). Normality of the distribution of variables was assessed using the Shapiro-Wilk test. Differences between the PV and control groups were compared using the nonparametric Mann-Whitney *U*-test. Correlation between variables of interest was evaluated using Spearman’s rank correlation coefficient. Assessment of statistically increased or decreased clonotype numbers was employed using one-tailed Fisher’s exact test, whereas for the statistical analysis of disease-associated clonotypes and probability of generation, the VDJRec pipeline was used. All statistical tests were two-tailed, and the results were considered statistically significant at a P-value < 0.05.

### Supplementary Information

Supplementary Figure S1.

Supplementary Tables.

Supplementary Table S5.

Supplementary Table S6.

## Data Availability

All sequencing data are available at the SRA repository (NCBI), under the BioProject PRJNA1026118 (http://www.ncbi.nlm.nih.gov/bioproject/1026118), with accession numbers from SAMN37734605 to SAMN37734624.
