# Supplementary material for: Characterization of the TCRβ repertoire of peripheral MR1-restricted MAIT cells in psoriasis vulgaris patients
Source: Sci Rep. 2023 Nov 28;13:20990. doi: 10.1038/s41598-023-48321-z (PMC10684872; doi:10.1038/s41598-023-48321-z)
Supplement: Supplementary file 1 — Supplementary Figure S1. [file 41598_2023_48321_MOESM1_ESM.pdf]

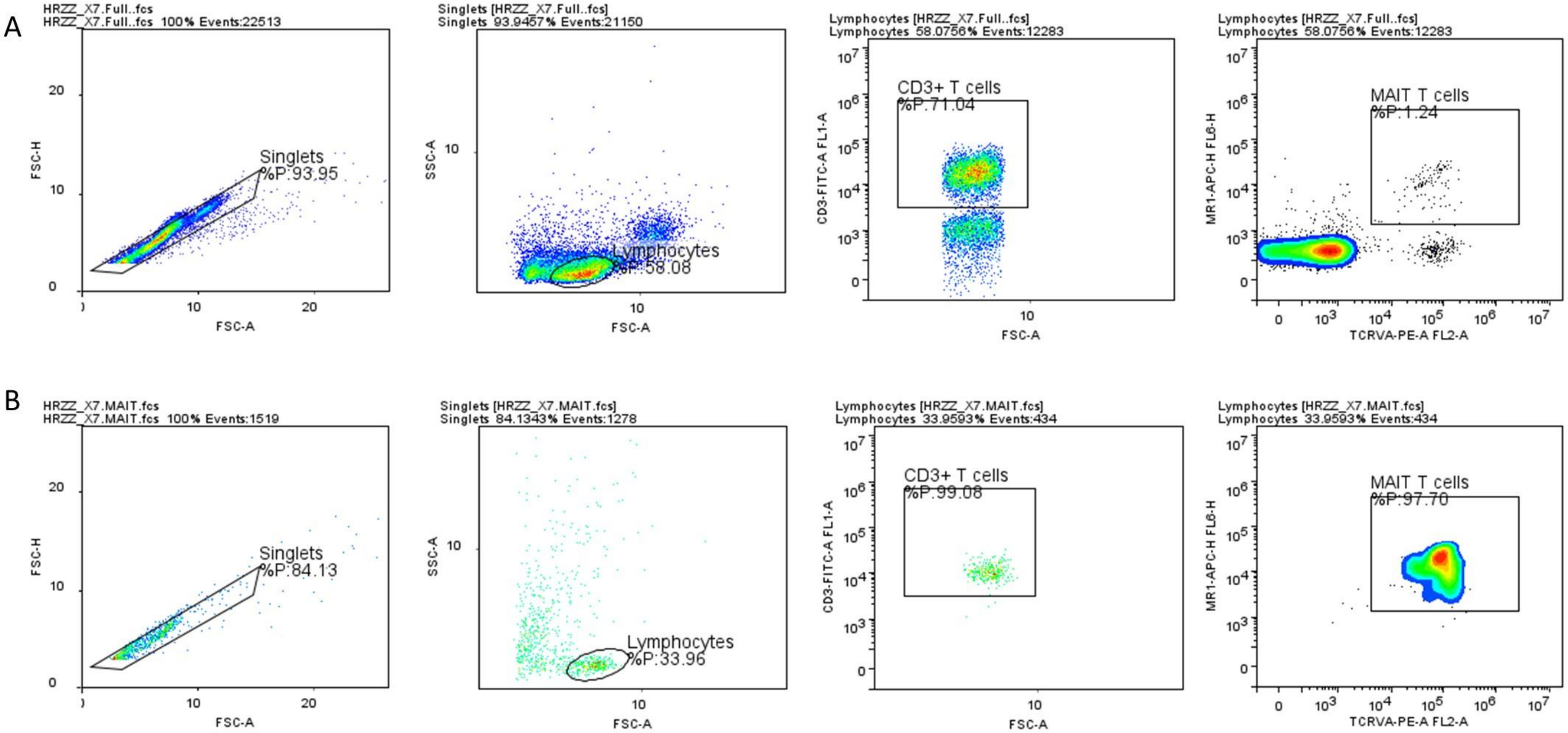

Supplementary Figure S1. Assessment of FACS sorting purity. (A) Fully stained sample ($CD3^{+}MR1\text{-}tet^{+}TCRV\alpha 7.2^{+}$) before FACS sorting, (B) Post-sort analysis of representative sample. Representative dot plots illustrate the gating strategy for MAIT cells identification. First, doublets were excluded by plotting forward scatter-area vs. forward scatter-height. The lymphocyte population was defined by size (forward scatter-area) and granularity (side scatter-area). Subsequently, T cells were selected as CD3-FITC-positive, and MAIT cells were identified as positive for MR1-tet-APC and TCRVα7.2- PE.
