# Supplementary material for: Characterization of the TCRβ repertoire of peripheral MR1-restricted MAIT cells in psoriasis vulgaris patients
Source: Sci Rep. 2023 Nov 28;13:20990. doi: 10.1038/s41598-023-48321-z (PMC10684872; doi:10.1038/s41598-023-48321-z)
Supplement: Supplementary file 2 — Supplementary Tables. [file 41598_2023_48321_MOESM2_ESM.pdf]

Supplementary Table S1. MAIT sorting results

Sample	Sorted MR1+ TCRVα7.2+ cells	Percentage of CD3+MR1-tet+ TCRVα7.2+ cells from total PBMCs (%)
C-1	49361	0.76
C-3	75827	2.51
C-4	120460	1.53
C-5	30000	0.68
C-6	209024	1.83
C-7	59331	1.49
C-9	61642	0.57
C-10	65901	1.32
PV-2	122725	1.49
PV-3	99525	0.72
PV-4	165900	1.37
PV-5	84552	0.84
PV-7	270744	2.04
PV-8	61690	0.53
PV-9	58713	0.7
PV-10	223096	2.53
Average	$1.10x10^5$	1.31
min	$3.00x10^4$	0.53
max	$2.71x10^5$	2.53

Supplementary Table S2. Sequencing run quality metrics.

Quality metric parameter	Score
Total reads	24557137
Reads identified (PF, %)	88.78
Cluster density (K/mm^2)	146 ± 2
Cluster PF (%)	78.76 ± 1.77
≥ Q30 base score (%)	82.85

*PF - passing filter

Supplementary Table S3. MiXCR analysis output

	PV	Controls	P*
Total sequencing reads	500745 (313611-590970)	343474 (278396-444052)	0.494
Successfully aligned reads (%)	86.6 (85.9-87.5)	87.3 (86.4-87.6)	0.293
Assembled to clones (%)	63 (57.8-64)	62 (60.8-64.3)	1.0
Final clonotype count	4582 (3243-5539)	3333 (2781-4873)	0.318
Average number of reads per clonotype	60.7 (50.4-75.6)	64.9 (44.8-73.9)	0.875

Data are shown as median (interquartile range). * Mann-Whitney U test, PV – psoriasis vulgaris

Supplementary Table S4. Results of VDJTools *Correct* function

Sample	Clonotypes before correction	Clonotypes after correction	Percentage of retained clonotypes
PV-2	4118	4053	98.42
PV-3	5942	5854	98.52
PV-4	10438	10248	98.18
PV-5	4900	4802	98
PV-7	2522	2486	98.57
PV-8	3113	3076	98.81
PV-9	2263	2221	98.14
PV-10	4233	4196	99.13
C-1	2993	2930	97.9
C-3	2603	2580	99.12
C-4	5303	5238	98.77
C-5	5335	5236	98.14
C-6	1621	1605	99.01
C-7	2942	2898	98.5
C-9	2393	2375	99.25
C-10	4214	4161	98.74
